# Proteomic Changes Associated With Sperm Fertilizing Ability in Meat-Type Roosters

**DOI:** 10.3389/fcell.2021.655866

**Published:** 2021-04-09

**Authors:** Anaïs Vitorino Carvalho, Laura Soler, Aurore Thélie, Isabelle Grasseau, Luiz Cordeiro, Daniel Tomas, Ana-Paula Teixeira-Gomes, Valérie Labas, Elisabeth Blesblois

**Affiliations:** ^1^CNRS, INRAE, Université de Tours, IFCE, Nouzilly, France; ^2^INRAE, ENVT, INP-Purpan, UPS, UMR Toxalim, Toulouse, France; ^3^INRAE, Université de Tours, CHU de Tours, Plate-forme PIXANIM (Phénotypage par Imagerie in/ex vivo de l’Animal à la Molécule), Nouzilly, France; ^4^INRAE, ISP, Université de Tours, Nouzilly, France

**Keywords:** sperm, chicken, proteomic, fertility, semen

## Abstract

The molecular basis of male fertility remains unclear, especially in chickens, where decades of genetic selection increased male fertility variability as a side effect. As transcription and translation are highly limited in sperm, proteins are key molecules defining their functionality, making proteomic approaches one of the most adequate methods to investigate sperm capacity. In this context, it is interesting to combine complementary proteomic approaches to maximize the identification of proteins related to sperm-fertilizing ability. In the present study, we aimed at identifying proteins related to fertility in meat-type roosters, showing fertility variability. Fertile roosters (fertility rates higher than 70% after artificial insemination) differed from subfertile roosters (fertility rates lower than 40%) in their sperm mass motility. Fertile and subfertile sperm protein contents were compared using two complementary label-free quantitative proteomic methods: Intact Cell MALDI-TOF-Mass Spectrometry and GeLC-MS/MS. Combining the two strategies, 57 proteins were identified as differentially abundant. Most of them were described for the first time as differentially abundant according to fertility in this species. These proteins were involved in various molecular pathways including flagellum integrity and movement, mitochondrial functions, sperm maturation, and storage in female tract as well as oocyte–sperm interaction. Collectively, our data improved our understanding of chicken sperm biology by revealing new actors involved in the complexity of male fertility that depends on multiple cell functions to reach optimal rates. This explains the inability of reductionist *in vitro* fertility testing in predicting male fertility and suggests that the use of a combination of markers is a promising approach.

## Introduction

Male fertility depends on the optimal functioning of all processes needed for sperm to fertilize the oocyte. The fertilizing ability of sperm is therefore reliant on the correct development and maturation of sperm cells including the acquisition of motility, structural integrity, viability, and its capacities to penetrate into the oocyte. Each aspect of these sperm functions are classically investigated by *in vitro* tests to diagnose fertility perturbations (WHO, 2010). However, these tests results are poorly correlated with *in vivo* male fertility in humans and in animals, suggesting the responsibility of more subtle dysfunctions of sperm biology (or putative combination of several suboptimal functions) in male subfertility, potentially revealed by molecular analysis (Soler et al., 2016a; Jodar et al., 2017).

Among the technical approaches available to investigate the molecular basis of male fertility, proteomics is particularly adequate (Kovac et al., 2013). Indeed, transcription and translation are very limited in mature sperm cells. Furthermore, proteins are considered as major effectors of cell functions (Jodar et al., 2017). Thus, sperm cell biology and activity are conditioned by the protein types, their abundance, and their posttranslational modifications at a given moment. Currently, mass spectrometry (MS)-based proteomics has been the most relevant approach to compare the sperm proteome from individuals with normal fertility and ones presenting difficulties to procreate in humans (Jodar et al., 2017; Bracke et al., 2018) and in animals (Park et al., 2013). Proteomic analysis of sperm cells provides two types of knowledge: on the one hand, global proteome analysis informs on the molecular pathways essential for sperm biology, and on the other hand, it helps identify potential male fertility biomarkers. These biomarkers are largely needed to predict the individual fertility capacity in human medicine (Jodar et al., 2017), animal reproduction (Druart et al., 2019), and genetic conservation programs of endangered breeds and species (Soler et al., 2020). Whereas each proteomic method provides a large amount of data and may propose potential biomarkers, due to the advantages and disadvantages of each methodology, a single approach cannot apprehend the complexities of male fertility. For instance, the Intact Cell MALDI-TOF Mass Spectrometry (ICM-MS) approach is a high-throughput methodology allowing the characterization of endogenous peptidoforms and proteoforms (<20 kDa) present in intact and whole cells without previous protein extraction or separation (Soler et al., 2020), at the difference of the bottom-up proteomic approach using a GeLC-MS/MS strategy allowing a larger overview of proteomes from extracted and digested protein samples.

Chicken is not only a popular laboratory model mainly for research on embryology, genetics, and behavior but also the first animal protein source for human consumption (FAO, 2020). Rooster fertility has decreased over the last decades in commercial breeds as a side effect of genetic selection especially for meat production (Rauw et al., 1998), but the molecular pathways involved in impaired sperm biology remain unclear. Previous investigations revealed that proteomic approaches applied to sperm cells are highly valuable to discriminate fertile and subfertile roosters (Labas et al., 2015; Soler et al., 2016a), but the molecular pathways involved in the sperm-fertilizing ability remains poorly described.

Here, to increase our understanding of subfertility causes in chickens, we first evaluated the semen quality from fertile and subfertile meat-type roosters in order to identify sperm defects relative to the fertility reduction observed in some animals. Furthermore, we used two complementary label-free quantitative proteomic approaches (ICM-MS and GeLC-MS/MS) at the individual level, in order to identify the proteins relative to sperm-fertilizing ability in chickens and potentially linked them to sperm quality defects and identify new cellular impairments not revealed by classical *in vitro* semen quality evaluation.

## Materials and Methods

### Animals

All experiments were carried out in accordance with the European welfare and the French Direction of Veterinary Services regulations (agreement number C37–175-1) as previously described (Soler et al., 2016a). Animal rearing was performed in the PEAT INRAe Poultry Experimental Facility (2018)^1^. Briefly, 360 adult ISABROWN females (ISA, Ploufragan, France) and 36 meat-line males acquired from Hubbard and Novogen (Quintin, France) were housed under a 14L/10D photoperiod and fed with standard diet of 12.5 MJ/day, supplemented with calcium for the females. Whereas males were housed in individual battery cages, hens were reared in groups of 5 animals.

### Semen Collection

Semen were collected twice a week by massage (Burrows and Quinn, 1937) for 5 weeks for *in vitro* analysis or *in vivo* fertility determination. After collection, sperm concentrations were immediately determined by light absorption (Accucell photometer, IMV Technologies, L’Aigle, France) at a wavelength of 530 nm (Brillard and McDaniel, 1985) and semen were diluted 1:1 in Beltsville Poultry Semen Extender (BPSE) (Sellier et al., 2006). Semen were used directly to analyze semen or inseminate females and aliquoted for proteomic investigation.

### *In vivo* Fertility Determination

To estimate the individual *in vivo* fertility of each male, 10 females per male were intravaginal artificially inseminated (AI) with a dose of 10^8^ sperm cells/female, two times at 1 week interval. Females were all fertile with almost one egg per day and randomly attributed to males. Fertility was determined by egg candling (from days 2 to 9 after the first AI and days 2–23 after the second AI). The fertility rate (FR) was defined as the percentage of fertile egg on the total number of analyzed egg (about 250 for each male). Males with FR >70% (*n* = 14) were considered fertile (F), whereas the subfertile (SF) population was associated with a FR <40% (*n* = 11) (Soler et al., 2016a). Nine animals per fertility status (F or SF) were randomly selected and included in this analysis. The difference of fertility between F and SF animals was explored by Mann–Whitney test, and significant difference was defined at 5% (*p* ≤ 0.05; R studio, R software version 4.0.2) (Allaire, 2015; R Core Team, 2017).

### *In vitro* Semen Analysis

As previously described (Labas et al., 2015; Soler et al., 2016a), semen quality was investigated with several *in vitro* parameters on F and SF animals. 7–8 ejaculates per rooster, collected in a period of 20 days, were included in the evaluation. Sperm volume was defined by weighing (ml), and sperm concentration was determined by light absorption (10^9^ sperm/ml), as previously described. Sperm viability (%) was determined with SYBR-14/propidium iodide fluorescent dyes, as previously described (Soler et al., 2016a), on at least 300 marked sperm per individual sample. Mass motility, defined as the movement of sperm group, was defined on a motility scale previously described (Blesbois et al., 2008): 0 as a total lack of movement and 9 as the presence of representing whirlwinds covering 30–60% of the observed area. Furthermore, objective measurements of motility were evaluated using the computer-assisted sperm analysis (CASA) system with an HTM-IVOS (Hamilton–Thorn Motility Analyzer, IVOS) (Blesbois et al., 2008). Thus, motility parameters such as straight-line velocity (VSL, μm/s), curvilinear velocity (VCL, μm/s), and average path velocity (VAP, μm/s) were individually determined as the mean of two semen samples. These parameters were also used to determine the percentages of motile, rapid, and progressive sperms as a motile sperm presented a VAP > 5 μm/s, rapid sperms showed a VAP > 50 μm/s, and a progressive cell was defined by a VAP > 50 μm/s. All preparations and observation were performed by the same observer. Each parameter was subjected to the Mann–Whitney test on the animal subset included in this analysis (9 animals F and 9 roosters SF; R studio, R software version 4.0.2) (Allaire, 2015; R Core Team, 2017). The statistical significance was defined at 5% (*p* ≤ 0.05).

### Intact Cell MALDI-TOF–Mass Spectrometry

Whole and intact sperm cells from F and SF roosters (*n* = 9) were analyzed by ICM-MS. Three ejaculated semen samples were collected from each male on BPSE as previously described. Sperm cells were isolated by centrifugation (600 × *g*, 10 min at 4°C). Sperm cells were washed twice with 1 ml of Tris Sucrose Buffer (TSB, 20 mM Tris–HCl, pH 6.8, and 260 mM sucrose) and then resuspended at 10^6^ sperm/μl. Using the dried droplet method, 1 μl of cell suspension was overlain with 2.5 μl of sinapinic acid matrix solution at 20 mg/ml dissolved in 50% acetonitrile/50% water in the presence of 2% trifluoroacetic acid. Twelve replicates were spotted for each sample onto an MTP Ground Steel 384 MALDI plate (Bruker Daltonics, Germany). The matrix/sample mix was allowed to evaporate at room temperature for 30 min. MS acquisitions were performed using a Bruker UltrafleXtreme MALDI-TOF instrument (Bruker Daltonics, Germany) equipped with a Smartbeam laser at a 2-kHz laser repetition rate following an automated method controlled by FlexControl 3.0 software (Bruker Daltonics, Germany). Spectra were obtained in positive linear ion mode in the m/z 1,000–20,000 range and collected from each spot as a sum of 1,000 laser shots in 5 shot steps (total of 5,000 spectra per spot). The parameters used for spectra acquisition were ion source 1, 25 kV; ion source 2, 23.55 kV; lens, 7 kV; pulsed ion extraction; and laser parameter set, large. Spectra were acquired three consecutive times per spot (36 technical replicates per sample). External calibration was applied using a mixture of known peptides and proteins as Glu1-fibrinopeptide B [M + H]^+^ = 1 571.59 m/z, ACTH (fragments 18–39) [M + H]^+^ = 2 466.68 m/z, insulin [M + H]^+^ = 5 734.52 m/z and ubiquitin [M + H]^+^ = 8 565.76 m/z, cytochrome C [M + H]^+^ = 12 360,97 m/z, and myoglobin [M + H]^+^ = 16 952.31 m/z. To increase mass accuracy (mass error < 0.05%), internal calibration was subsequently applied to all spectra. The latter was achieved by performing a lock mass correction using flexAnalysis 4.0 software (Bruker) with the mass of the highest-intensity peak in the middle of the mass range, corresponding to the protein phosphoglycerate kinase (7,983.457 m/z). All spectra were converted in format mzXML using the CompassXport tool (Bruker Daltonics) and treated using the free and open-source analysis software R (version 3.6.1) along with specific package for Quantitative Analysis of Mass Spectrometry Data as MALDIquant (v1.19.3) (Gibb and Strimmer, 2012). The spectra were treated using the square root intensities, and the baseline estimation was based on the TopHat filter (van Herk, 1992; Gil and Werman, 1993). Spectra were smoothed using the Savitzky–Golay filter, realigned using prominent peaks, and normalized on intensity using the total ion current (TIC) method (Filzmoser and Walczak, 2014). Peaks were detected using the Median Absolute Deviation (MAD) method with a signal-to-noise ≥ 2. To retain only quality spectra for each sperm sample, the dataset (*n* = 36 technical spectra) was filtered by correlation analyses by comparing the spectra two by two. Then, we computed filter-based correlation coefficients with iterations from 40 to 90% for each technical spectrum vs. all other spectra of the same class. All retained spectra were merged to generate a total average spectrum for each sample. The *x*-axis of spectra represented the m/z values of biomolecules, and the *y*-axis represented the normalized peak intensities.

The intra-assay precisions of the ICM-MS acquisitions were determined by calculating the coefficient of variation (% CV) of the normalized peak-intensity values from the technical replicates for each ejaculate (*n* = 27). The CV% are ranged from 16.71 to 46.88% with a mean CV at 29.41%. The inter-assay precision was determined by calculating the % CV of the mean peak-normalized values for the three ejaculates of all animals. For F and SF populations, the mean% CV did not exceed 36.76 and 36.38%, respectively.

The MS data did not pass the normality and homogeneity QQ plot and Kolmogorov–Smirnov tests. Therefore, for comparative analyses, data were submitted to the non-parametric Mann–Whitney or Wilcoxon test to characterize changes between the two fertility conditions. M/z were considered significantly differential when a *p* < 0.05 and a fold-change ratio (FC) ≤ 0.66 or ≥ 1.5. Principal component analysis (PCA) and volcano plot were performed using the gplots (v3.0.3) and FactoMineR (v2.1) packages of the R software (R Core Team, 2017).

The peaks observed by ICM-MS were annotated using a home database generated from *Gallus gallus* sperm top-down proteomics, containing ∼800 biomolecules already identified in chicken spermatozoa (Soler et al., 2016a,b). The ICM-MS peaks were aligned to theoretical peptidoform and proteoform average masses [M + H]^+^ considering their posttranslational modifications, with a mass tolerance ≤ 0.05% (± 500 ppm) (Supplementary Data 1).

### Bottom-Up Proteomic by GeLC-MS/MS

Four animals from each fertility status (SF and F) were randomly selected to be analyzed by a bottom-up proteomic approach combining SDS-PAGE fractionation, in-gel protein digestion by trypsin, and nano-Liquid Chromatography coupled to high-resolution tandem mass spectrometry (GeLC-MS/MS). Briefly, proteins were extracted as previously described (Labas et al., 2015) and 50 μg of total protein was submitted to SDS-PAGE (4–20%, 8.3 × 6 cm × 1.5 mm). After Coomassie Blue staining, each gel lane was fractionated into 11 slices. Gel pieces were washed in water with acetonitrile (1:1), then cysteine reduction and alkylation were performed by successive incubations with 10 mM dithiothreitol in 50 mM NH_4_HCO_3_ (30 min, at 56°C) and 55 mM iodoacetamide in 50 mM NH_4_HCO_3_ (20 min, at room temperature, in the dark). Digestion was carried out overnight using 25 mM NH_4_HCO_3_ with bovine trypsin (sequencing grade, Roche Diagnostics, Paris, France) at 12.5 ng/μl. Peptides were extracted and dried using a SPD1010 speedvac system (ThermoSavant, Thermo Fisher Scientific). For each protein band, the resultant peptide mixture was reconstituted with 30 μl of 0.1% formic acid and 2% acetonitrile, sonicated for 10 min and analyzed by nanoLC-MS/MS in triplicate. Proteomic experiments were performed using an LTQ Orbitrap Velos mass spectrometer coupled to an Ultimate^®^ 3000 RSLC Ultra High-Pressure Liquid Chromatographer (Thermo Fisher Scientific, Bremen, Germany) as previously described (Labas et al., 2015). A peptide mixture (5 μl) was loaded on a trap column for desalting and separated using a nano-column. The gradient consisted of 4–55% B for 90 min at a 300 nl/min flow rate. The mass spectrometer was operated in positive data-dependent mode using Xcalibur software (version 2.1; Thermo Fisher Scientific, San Jose, CA). In the scan range of m/z 300–1,800 with a targeted resolution at 60,000, the 20 most intense peptide ions with charge states ≥ 2 were fragmented using Collision Induced Dissociation (CID). A lock mass corresponding to polydimethylcyclosiloxane ions [m/z, 445.1200025 (Si(CH_3_)_2_O)_6_] was enabled for accurate mass measurements.

Protein data identification was performed using Mascot search engine version 2.7.0.1 (Matrix Science, London, United Kingdom) combined to Proteome Discoverer 2.1.1.21 software (Thermo Fisher Scientific, Bremen, Germany), against the chordata section of the non-redundant NCBI database (download august 2020). The search parameters included trypsin as a protease with two allowed missed cleavages and carbamidomethylcysteine, methionine oxidation, and acetylation of N-term protein as variable modifications. The tolerance of the ions was set to 5 ppm for parent and 0.8 Da for fragment ion matches. Mascot results obtained from the target and decoy databases searches were subjected to Scaffold software (v 4.11.1, Proteome Software, Portland, United States) using the protein cluster analysis option (assemble proteins into clusters based on shared peptide evidence). Peptide and protein identifications were validated and accepted if they could be established at greater than 95.0% probability as specified by the Peptide Prophet algorithm (Keller et al., 2002) and by the Protein Prophet algorithm (Nesvizhskii et al., 2003), respectively. Protein identifications were accepted if they contained at least two identified peptides. The correspondent% decoy false discovery rate (FDR) done by Scaffold software was < 0.0% regarding the number of protein/clusters, and FDR was < 0.00% for the 141,914 spectra, which was in accordance with FDR values acceptable by Mass Spectrometry Data Interpretation Guidelines (<1%) (Deutsch et al., 2016). Quantifications were based on a label-free approach using two independent quantitative methods: the Spectral Counting (SC) using the “Weighed Spectra” method (Liu et al., 2004) and the Average Precursor Intensity (API) method (Cox et al., 2014). A *t*-test was performed to characterize changes between F and SF roosters. Differences were considered statistically significant for *t*-test with a *p* < 0.05. Limits of an average normalized weighted spectra (NWS) of ≥ 5 and fold change of ≤ 0.71 or ≥ 1.4 were included to increase the validity of comparison. MS data have been deposited to the ProteomeXchange Consortium via the PRIDE partner repository (Vizcaíno et al., 2014) with the dataset identifier PXD022322 and 10.6019/PXD022322.

For each significant cluster, one significant protein with an annotation (exclusion of LOC proteins without gene symbol and defined by their gene loci), a unique gene symbol, and a gene description has been randomly chosen to represent the cluster (*n* = 39, Supplementary Data 2). When the unique gene was defined as significant by SC and API methods, the SC analysis was kept. The mean signal of each differentially abundant protein (DAP) with unique gene symbol for each individual was calculated based on the three technical replicates by animal (Supplementary Data 2). Data exploration was performed with R (version 4.0.2) (R Core Team, 2017). Protein abundances obtained by GeLC-MS/MS, mass motility, and fertility contributions to the sample variability were explored by principal component analysis (PCA) with the package factoextra (version 1.0.7).

### Western Blotting

Sperm cells were separated by centrifugation (600 × *g*, 10 min, 20°C) from the seminal plasma for 4 F and 4 SF males randomly selected. Sperm proteins were extracted by sonication in lysis buffer (150 mM NaCl, 10 mM Tris–HCl, 1 mM EGTA, 1 mM EDTA, 200 mM sodium fluoride, 4 mM sodium pyrophosphate, 2 mM sodium orthovanadate, 1% Triton X-100, and 0.5% NP40) with protease inhibitor EDTA-free (1 tablet/10 ml buffer, 04693159001, Roche Diagnostics, Mannheim, Germany). Twenty-five micrograms of protein was included in gradient 8–16% gradient SDS-PAGE, before being transferred onto nitrocellulose membranes (Amersham Protran 0.2 μm NC, 10-600001, GE Healthcare Life Science, Germany). Total protein staining on membranes was achieved using Revert^TM^ 700 Total Protein Stain (cat. 926-11011, LI-COR Biosciences, United States) according to the manufacturer’s instructions and scanned with the Odyssey CLx Imaging System (LI-COR Biosciences, Germany). Membranes were incubated with primary antibody overnight at 4°C, then incubated with the appropriate fluorescent-conjugated secondary antibody, IRDye 800CW goat anti-rabbit (cat. 926-32211, LI-COR Biosciences, United States) or IRDye 800CW goat anti-mouse (cat. 926-32210, LI-COR Biosciences, United States), diluted 1:2,000 in Odyssey blocking buffer (cat. 927-50000, LI-COR Biosciences, United States) diluted 1:1 (v:v) in Tris-buffered saline buffer (TBS) (cat. N14581, Interchim Life Sciences, France). The optimized primary antibodies were used as follows: rabbit anti-lysozyme (1:500) and mouse anti-ovotransferrin [1:1,000, mouse (Gautron et al., 2001)] in 0.1% Tween 20 TBS (TBS, cat. N14581, Interchim Life Sciences, France; Tween-20, cat. P9516, Sigma, Germany) with 5% (w/v) non-fat dry milk, at room temperature. The antibodies raised against lysozyme and ovotransferrin were a kind gift from S. Réhault-Godbert and J. Gautron (INRAE, University of Tours, BOA, Nouzilly, France). It was produced by ProteoGenix (Schiltigheim, France) after immunization of rabbits with purified egg white lysozyme (Sigma-Aldrich, Saint-Quentin-Fallavier, France). A protein-specific band signal was normalized against total protein stain signal, and differences were evaluated using a Mann–Whitney test (R studio, R software version 4.0.2) (Allaire, 2015; R Core Team, 2017). The statistical significance was defined at 5% (*p* < 0.05).

## Results

### Fertility and *in vitro* Characterization of Semen

**FIGURE 1:**
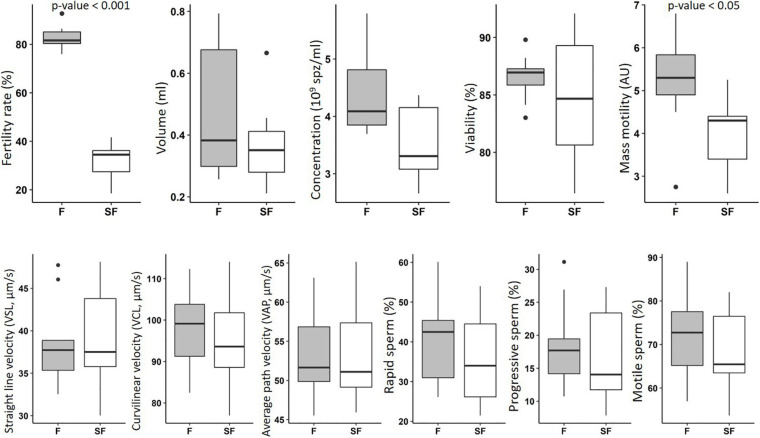
*In vitro* characterization of semen from fertile (F) and subfertile (SF) meat-line chickens. Semen samples (9 roosters per fertility status) were subjected to *in vitro* evaluation including the determination of volume, concentration, viability, and several motility parameters. F animals in gray; SF animals in white. Data were analyzed with Mann–Whitney test and statistical difference was defined with *p* ≤ 0.05.

The *in vitro* characterization of the semen from F and SF roosters included the investigation of volume, concentration, viability, and several motility parameters. The significant decrease of *in vivo* fertility observed in SF animals is associated with a reduction of mass motility (Figure 1). No significant impact was observed on the other *in vitro* parameters investigated, i.e., volume, concentration, viability, and other motility parameters, obtained with CASA analysis.

### Comparison of Sperm ICM-MS Profiles From F and SF Roosters

**FIGURE 2:**
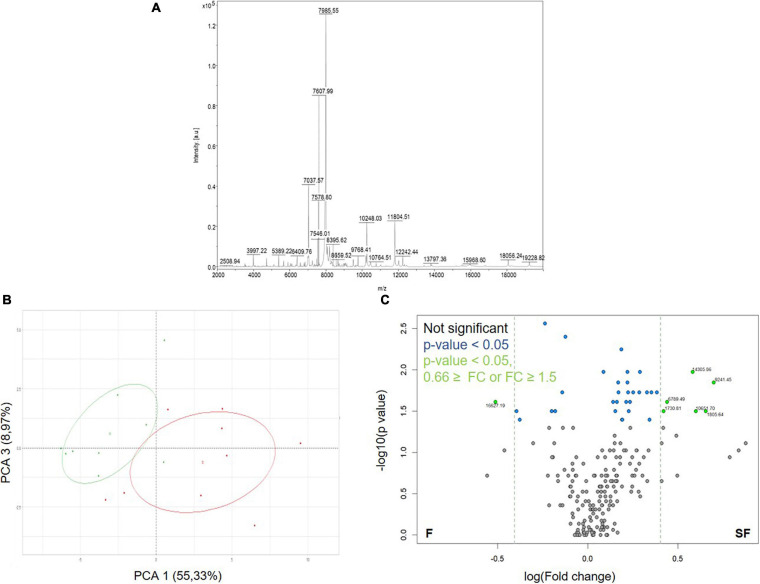
Protein content exploration of sperm from fertile (F) and subfertile (SF) meat-line roosters by ICM-MS. **(A)** Representative sperm cell ICM-MS spectra from one F animal. **(B)** Principal component analysis (PCA) of ICM-MS signals obtained for F (in green) and SF (in red) roosters. **(C)** Volcano plot analysis between F and SF animals of detected m/z peaks (*n* = 211). Gray points indicate not significant peaks, blue ones, peaks with a significant abundance difference between F and SF animals (*p* < 0.05), and green ones, the significant peaks presenting a fold change (FC) ≤ 0.66 or ≥ 1.5.

We firstly applied the ICM-MS approach to sperm sampled from F and SF roosters (Figure 2 and Supplementary Data 1). Sperm ICM-MS profiling from meat-line roosters revealed 211 detected peaks in total (Figure 2A). Descriptive analysis performed by PCA reported a clear segregation of F and SF samples based on PCA1 (55.33%) and PCA3 (8.97%) (Figure 2B). The differential analysis revealed 36 m/z peaks differentially abundant between F and SF animals (*p* < 0.05, Figure 2C). Among them, 7 presented a fold-change ratio (FC) ≤ 0.66 or ≥ 1.5 (Figure 2C). The annotation of these peaks was performed by computational confrontation against a homemade database of chicken sperm proteoforms and peptidoforms identified by Top-Down MS (Soler et al., 2016a). Three differential peaks with low m/z corresponding to peptidoforms (as results of protein degradation) were reported: cilia- and flagella-associated protein 100 (CFAP100, 1730.81 m/z, NPFTIPPDIDIFAIR), alpha enolase (ENO1, 1805.64 m/z, AAVPSGASTGIYEALELR) and tubulin beta-7 chain (TUBB7, 6789.49 m/z, SGPFGQIFRPDNFVFGQ SGAGNNWAKGHYTEGAELVDSVLDVVRKEAESCDCLQGF QLTHSLG). Manual annotation allowed to describe one whole protein: Lysozyme (LYZ, 14305.86 m/z). These three peptidoforms and one protein were more abundant in SF sperm cells than in F samples (Figure 2C).

### Comparison of Sperm Proteome From F and SF Roosters by GeLC-MS/MS Analysis

To increase the number of identified DAP and obtain a global overview of semen proteome, we applied a bottom-up proteomic approach and compared F and SF sperm cell profiles. The GeLC-MS/MS investigation of sperm protein content of F and SF animals allowed the detection of 745 proteins included in 327 clusters. Among them, 27 and 50 DAP were identified by the SC and API quantitative methods, respectively, and 7 were comforted by both approaches. Consequently, a total of 84 DAP were identified with differential abundance between F and SF roosters by GeLC-MS/MS analysis (Table 1).

**TABLE 1:** Differentially abundant proteins between fertile (F) and subfertile (SF) meat-line chickens identified by GeLC-MS/MS.

					**Spectral counting**		**API**	
**#**	**Gene description**	**Accession number**	**Molecular weight (kDa)**	**Gene symbol**	***p*-value**	**F/SF FC**	***p*-value**	**F/SF FC**
1.51	Tubulin beta-1 chain isoform X2 [*Electrophorus electricus*]	XP_026866564.1	49	TUBB1	0.240	0.80	**0.029**	**0.70**
1.53	Beta tubulin, partial [*Gillichthys mirabilis*]	AAL24510.1	17		0.087	0.70	**0.011**	**1.90**
2.35	Tubulin alpha-2 chain isoform X2 [*Numida meleagris*]	XP_021246036.1	43	LOC110395670	0.380	1.30	**0.039**	**0.60**
2.38	Tubulin alpha-8 chain-like [*Cebus capucinus imitator*]	XP_017390722.1	53	LOC108309094	0.210	0.80	**0.043**	**0.70**
2.53	Tubulin alpha-8 chain-like [*Sphaeramia orbicularis*]	XP_029994229.1	51	LOC115422205	0.030	0.50	**0.005**	**0.40**
6.4	Hexokinase-3 isoform X1 [*Coturnix japonica*]	XP_015731713.1	109	HK3	0.700	1.10	**0.002**	**1.90**
6.6	Hexokinase-3-like [*Tyto alba alba*]	XP_032839590.1	105	LOC104357108	0.091	6.80	**0.049**	**2.60**
7.25	Hypothetical protein A6R68_14483, partial [*Neotoma lepida*]	OBS74971.1	39		0.009	2.80	**0.016**	**3.30**
8.1	Fructose-bisphosphate aldolase A isoform X2 [*Equus caballus*]	XP_003362760.1	39	ALDOA	0.065	1.10	**0.010**	**1.60**
8.1	Fructose-bisphosphate aldolase C, partial [*Lepidosiren paradoxa*]	BAD17883.1	36		**0.019**	**2.80**	0.170	1.40
8.14	Fructose-bisphosphate aldolase A-like, partial [*Apteryx rowi*]	XP_025921567.1	29	LOC112966083	**0.019**	**1.80**	0.100	1.20
8.2	Fructose-bisphosphate aldolase A [*Corvus moneduloides*]	XP_031953954.1	39	ALDOA	0.027	1.30	**0.004**	**1.50**
8.3	Fructose-bisphosphate aldolase A-like [*Meleagris gallopavo*]	XP_019467335.1	8	LOC104917114	0.096	1.20	**0.003**	**1.70**
8.4	Fructose-bisphosphate aldolase A isoform X1 [*Desmodus rotundus*]	XP_024416190.1	45	ALDOA	0.200	1.10	**0.003**	**1.80**
8.5	Fructose-bisphosphate aldolase A-like [*Numida meleagris*]	XP_021239723.1	14	LOC110392092	0.140	1.10	**0.043**	**1.50**
8.6	Aldolase, fructose-bisphosphate A S homeolog [*Xenopus laevis*]	NP_001079649.1	39	ALDOA	0.340	1.10	**0.006**	**1.70**
8.7	Fructose-bisphosphate aldolase A [*Pogona vitticeps*]	XP_020654424.1	40	ALDOA	0.150	1.20	**0.042**	**1.50**
8.8	Fructose-bisphosphate aldolase A, partial [*Pygoscelis adeliae*]	KFW62523.1	17	LOC103925596	**0.046**	**1.40**	0.150	1.20
8.9	Fructose-bisphosphate aldolase C [*Coturnix japonica*]	XP_015736436.1	39	ALDOC	**0.024**	**1.50**	**0.017**	**1.50**
10.1	Creatine kinase B-type [*Gallus gallus*]	NP_990641.1	43	CKB	**0.011**	**1.50**	**0.046**	**1.50**
10.11	Creatine kinase B-type isoform X1 [*Nanorana parkeri*]	XP_018412106.1	43	CKB	0.420	1.30	**0.044**	**1.60**
10.13	Creatine kinase M-type [*Papio anubis*]	XP_003915756.1	43		0.094	2.00	**0.003**	**6.20**
10.2	Creatine kinase B-type isoform X1 [*Gallus gallus*]	XP_015142790.1	42	CKB	**0.043**	**1.40**	0.055	1.50
10.3	Creatine kinase B-type isoform X1 [*Apteryx mantelli mantelli*]	XP_013796252.1	43	CKB	**0.013**	**1.40**	0.160	1.30
10.5	Creatine kinase, brain, isoform CRA_a [*Homo sapiens*]	EAW81820.1	43	CKB	**0.020**	**1.40**	0.790	0.90
10.6	Creatine kinase B-type isoform X1 [*Chaetura pelagica*]	XP_010006458.1	43	CKB	**0.011**	**1.70**	0.150	1.50
10.9	Creatine kinase B-type [*Mesocricetus auratus*]	XP_005068378.1	43	CKB	**0.048**	**1.40**	0.330	0.70
13.3	Hypothetical protein CIB84_011751 [*Bambusicola thoracicus*]	POI24503.1	12		0.160	1.20	**0.010**	**1.70**
14.1	Ovotransferrin [*Gallus gallus*]	P02789.2	78	TF	**0.028**	**1.40**	0.260	1.40
14.2	Ovotransferrin BC type [*Gallus gallus*]	BAE16339.1	78	TF	**0.042**	**1.40**	0.390	1.30
18.1	ATP synthase subunit alpha, mitochondrial, partial [*Colius striatus*]	XP_010196224.1	58	ATP5F1A	**0.001**	**0.70**	0.760	1.00
18.5	ATP synthase subunit alpha, mitochondrial-like [*Arvicanthis niloticus*]	XP_034353318.1	60	LOC117705093	0.005	0.60	**0.008**	**1.60**
22.1	Keratin 1 [*Homo sapiens*]	AAG41947.1	66	KRT1	**0.019**	**1.60**	0.095	1.80
22.3	Keratin, type II cytoskeletal 1 isoform X2 [*Macaca mulatta*]	XP_001098182.2	65	KRT1	**0.034**	**2.10**	0.360	1.40
22.8	Keratin, type II cytoskeletal 6A isoform X2 [*Pongo abelii*]	XP_024113011.1	57	LOC100449906	**0.002**	**4.30**	**0.012**	**2.70**
24.1	Heat shock protein Hsp70 [*Gallus gallus*]	AAN18280.1	70	HSPA2	**0.045**	**1.60**	0.750	1.10
24.7	Heat shock 70 kDa protein [*Gallus gallus*]	BBO36590.1	70	HSPA2	**0.024**	**2.00**	0.910	1.00
27.2	Hypothetical protein N333_09691, partial [*Nestor notabilis*]	KFQ42654.1	45	GOT2	0.530	0.90	**0.005**	**1.70**
32.1	Hypothetical protein ASZ78_016751 [*Callipepla squamata*]	OXB67628.1	45		**0.003**	**1.80**	0.140	1.30
32.11	Phosphoglycerate kinase 1 [*Paralichthys olivaceus*]	XP_019941634.1	44	PGK1	0.110	1.80	**0.040**	**1.80**
32.2	Phosphoglycerate kinase 1 [*Ceratotherium simum simum*]	XP_004442122.1	45	LOC101387600	**0.001**	**1.60**	**0.010**	**1.80**
32.3	Phosphoglycerate kinase 1 [*Echeneis naucrates*]	XP_029374590.1	44	PGK1	0.020	1.60	**0.045**	**1.50**
32.8	Phosphoglycerate kinase 2-like [*Ictidomys tridecemlineatus*]	XP_005318639.1	45	LOC101964802	0.002	1.60	**0.014**	**1.70**
33.1	Keratin 10 [*Homo sapiens*]	AAH34697.1	59	KRT10	**0.010**	**1.50**	0.900	1.00
33.2	Keratin, type I cytoskeletal 9 [*Homo sapiens*]	NP_000217.2	62	KRT9	**0.022**	**2.30**	0.170	2.00
33.3	Keratin 14 [*Homo sapiens*]	AAH02690.1	52	KRT14	**0.000**	**4.20**	0.180	1.70
46	Unnamed protein product, partial [*Gallus gallus*]	CAA30161.1	54	LOC107051274	**0.000**	**0.30**	0.400	0.80
49.1	Mitochondria-eating protein isoform X1 [*Gallus gallus*]	XP_004936087.1	62	SPATA18	**0.001**	**1.60**	0.094	1.40
56.1	Citrate synthase, mitochondrial isoform X2 [*Gallus gallus*]	XP_015155774.1	56	CS	**0.020**	**1.40**	**0.003**	**1.90**
59	Hypothetical protein CIB84_000737 [*Bambusicola thoracicus*]	POI35507.1	30		**0.013**	**1.60**	0.420	1.20
60.1	Cytochrome c [*Gallus gallus*]	NP_001072946.1	12	CYCS	**0.037**	**0.60**	0.310	1.20
63.1	Spermatid-specific manchette-related protein 1 isoform X2 [*Gallus gallus*]	XP_004937159.1	30	CZH9orf24	**0.030**	**0.30**	0.180	0.60
65.1	Complex I assembly factor ACAD9, mitochondrial [*Gallus gallus*]	NP_001006136.1	68	ACAD9	**0.009**	**1.60**	0.980	1.00
65.2	Acyl-CoA dehydrogenase family member 9, mitochondrial [*Rhincodon typus*]	XP_020383709.1	70	ACAD9	0.015	3.00	**0.032**	**4.60**
68.4	Hypothetical protein parPi_0021429 [*Paroedura picta*]	GCF58429.1	47		0.580	0.90	**0.047**	**1.50**
69.3	Long-chain specific acyl-CoA dehydrogenase, mitochondrial [*Monopterus albus*]	XP_020470903.1	50	ACADL	0.430	1.20	**0.039**	**1.40**
82.3	Ornithine aminotransferase, mitochondrial [*Phaethon lepturus*]	XP_010295191.1	48	OAT	0.013	2.20	**0.038**	**1.70**
89.1	T-complex protein 1 subunit zeta [*Gallus gallus*]	NP_001006216.1	58	CCT6A	**0.014**	**1.50**	0.540	1.20
89.4	T-complex protein 1 subunit zeta [*Phascolarctos cinereus*]	XP_020862612.1	58	CCT6A	0.070	2.20	**0.004**	**3.60**
91.2	Superoxide dismutase [Mn], mitochondrial, partial [*Thamnophis sirtalis*]	XP_013926038.1	18	SOD2	0.072	0.80	**0.039**	**0.70**
92.1	T-complex protein 1 subunit alpha, partial [*Eudyptula novaehollandiae*]	KAF1505402.1	62		0.710	1.10	**0.044**	**1.60**
92.2	Thermosome subunit beta [*Turdus rufiventris*]	KAF4796296.1	48		0.220	1.40	**0.032**	**1.50**
92.3	PREDICTED: T-complex protein 1 subunit alpha [*Anolis carolinensis*]	XP_003215812.1	60	TCP1	0.560	1.20	**0.033**	**1.60**
93.1	Hypothetical protein RCJMB04_35g11 [*Gallus gallus*]	CAG32769.1	55	LOC107056139	0.130	2.00	**0.022**	**2.40**
98.1	Tektin-3 [*Gallus gallus*]	XP_004946137.1	56	TEKT3	0.067	0.00	**0.031**	**0.00**
103.1	Peroxisomal carnitine O-octanoyltransferase [*Gallus gallus*]	XP_003640711.1	70	CROT	0.400	1.30	**0.019**	**2.50**
135.1	T-complex protein 1 subunit delta [*Gallus gallus*]	NP_996761.1	58	CCT4	0.590	1.20	**0.035**	**4.00**
135.2	T-complex protein 1 subunit delta-like [*Trachypithecus francoisi*]	XP_033033221.1	58	LOC117063540	0.100	2.00	**0.002**	**3.10**
138.1	Tektin-4 [*Gallus gallus*]	XP_414831.1	53	TEKT4	0.051	0.20	**0.012**	**0.20**
139	Dynein light chain LC6, flagellar outer arm [*Gallus gallus*]	XP_001233550.2	10	DYL2	0.180	1.60	**0.008**	**3.50**
148.1	Lysozyme [*Gallus gallus*]	P00698.1	16	LYZ	**0.001**	**0.30**	**0.001**	**0.40**
153	Apolipoprotein A-I [*Gallus gallus*]	AAA48597.1	31	APOA1	**0.008**	**0.50**	0.510	0.90
178.1	cAMP and cAMP-inhibited cGMP 3’,5’-cyclic phosphodiesterase 10A isoform X1 [*Gallus gallus*]	XP_004935608.1	116	PDE10A	0.260	1.60	**0.034**	**2.80**
187	Transthyretin isoform 2 [*Gallus gallus*]	NP_001268427.1	19	TTR	**0.013**	**0.60**	**0.025**	**0.70**
217.1	Plasminogen [*Gallus gallus*]	XP_419618.2	91	PLG	0.006	0.00	**0.015**	**0.00**
224.1	Hypothetical protein N338_03343, partial [*Podiceps cristatus*]	KFZ67843.1	43		0.007	2.90	**0.001**	**2.70**
253.1	Annexin A1 [*Gallus gallus*]	NP_996789.1	39	ANXA1	0.031	0.03	**0.030**	**0.00**
263	Hypothetical protein ASZ78_013233 [*Callipepla squamata*]	OXB59879.1	23		0.021	2.10	**0.007**	**2.10**
264	Immunoglobulin heavy chain variable region, partial [*Gallus gallus*]	BAA11105.1	11		0.002	0.40	**0.000**	**0.30**
275.1	Nucleoside diphosphate kinase homolog 5 [*Gallus gallus*]	NP_001244300.2	24	NME5	0.092	0.20	**0.048**	**0.20**
284.1	Acetyl-CoA acetyltransferase, cytosolic [*Gallus gallus*]	NP_001034376.2	41	ACAT2	0.058	4.20	**0.027**	**6.10**
293	Alpha-2-HS-glycoprotein [*Gallus gallus*]	XP_422764.1	37	AHSG	0.028	0.00	**0.014**	**0.00**
310.1	Disintegrin and metalloproteinase domain-containing protein 32-like [*Gallus gallus*]	XP_024998701.1	82	ADAM32	0.027	4.20	**0.026**	**3.50**
311.1	Hypothetical protein CIB84_002892 [*Bambusicola thoracicus*]	POI33356.1	51		0.019	0.00	**0.025**	**0.00**

**Significant abundance differences were characterized between F and SF animals by applying t-test and retaining only accession numbers with p-values ≤ 0.05 associated with a fold-change ratio (FC) ≤ 0.71 or ≥ 1.4. Significant peptides are indicated with bold characters within columns corresponding to spectral counting and API (Average Precursor Intensity) quantitative methods. #: number of the considered cluster (before the point) and peptides (after the point).**

**FIGURE 3:**
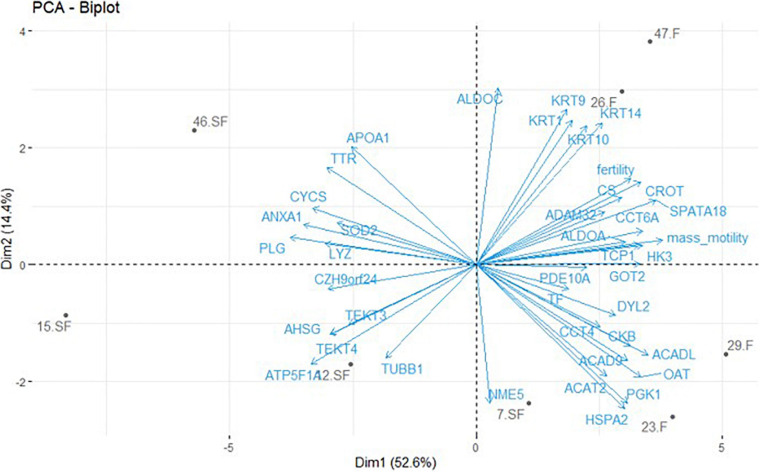
Principal component analysis (PCA) of protein abundances obtained by GeLC-MS/MS with unique gene identification combined with fertility status and semen mass motility. Only DAP with a unique gene and an annotation (exclusion of LOC proteins) were included in this analysis. One significant DAP per cluster was retained and the mean signal calculated based on the three technical replicates obtained for each animal. Animals are revealed by their identification number and their fertility status (F = fertile; SF = subfertile). Animals are represented in gray and protein contribution as well as semen parameters in blue.

A list of unique genes was developed based on DAP with a gene description and a gene symbol corresponding to the described protein (i.e., DAP list obtained from GeLC-MS/MS without LOC proteins, *n* = 39) (Supplementary Data 2). This list was used for functional analysis. Combined with animal fertility status and semen mass motility, the PCA analysis (Figure 3) revealed that the first dimension (52.6% of the variability) represented animals clustering according to their fertility status and their sperm mass motility. According to this dimension, proteins with unique genes and annotation were speared by their expression fold change: with negative values, the more abundant proteins in SF roosters, and with positive value, the ones more abundant in F roosters. Dimension 2 only represented 14.4% of the variability (Figure 3) and preferentially represented interindividual variability within a fertility status.

### Western Blotting

**FIGURE 4:**
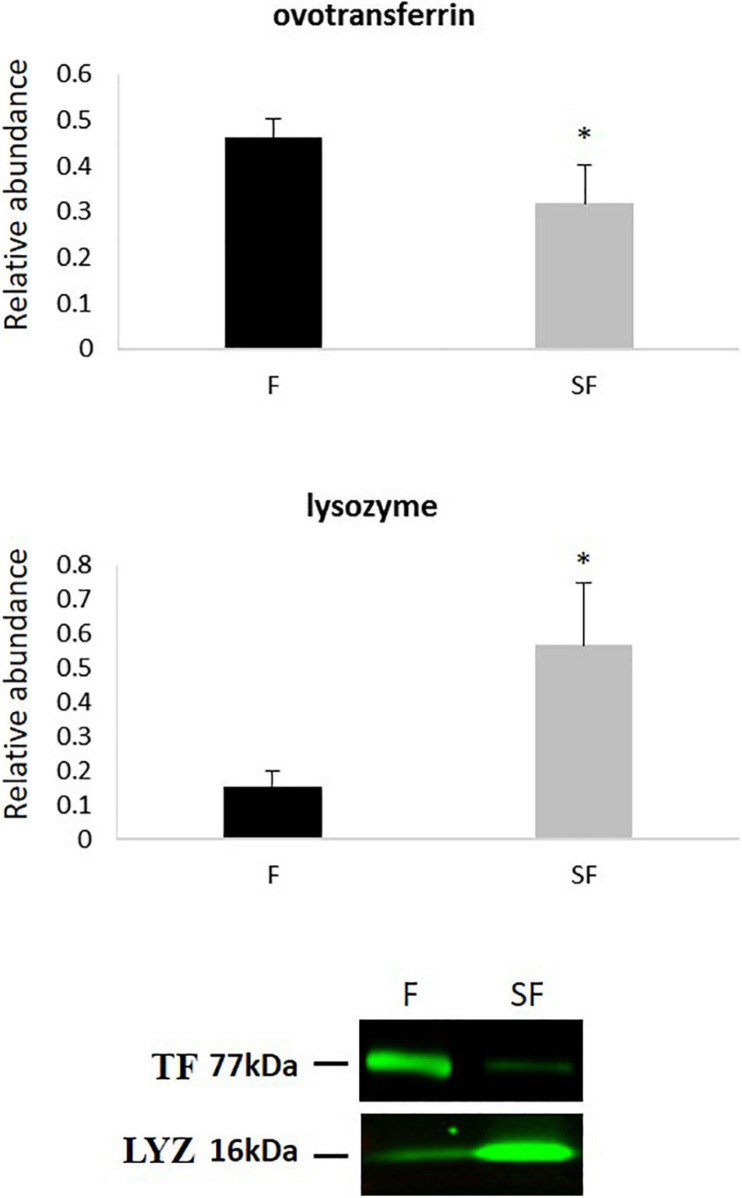
Western-blot quantification and signals of ovotransferrin (TF) and lysozyme (LYZ) in sperm from fertile (F) and subfertile (SF) roosters. *p < 0.05.

The differential abundance of ovotransferrin (TF) and lysozyme (LYZ) was validated by western blotting. Both proteins presented a significant differential abundance between F and SF sperm, and abundance changes were in the same direction as in the proteomic analysis (Figure 4 and Table 2). A decrease of TF was revealed in SF samples by western blotting as well as by GeLC-MS/MS, and LYZ was more abundant in SF animals in western blotting as well as in ICM-MS, GeLC-MS/MS approaches (Figure 4 and Table 2).

**TABLE 2:** Comparison of the protein abundance fold change between fertile (F) and subfertile (SF) sperm obtained by ICM-MS, GeLC-MS/MS, and western blotting for ovotransferrin and lysozyme.

	**Fold change (F/SF)**		
**Protein name**	**ICM-MS**	**GeLC-MS/MS (spectral counting)**	**Immunoblotting**
Ovotransferrin	/	1.4	1.5
Lysozyme	0.5	0.3	0.3

## Discussion

The molecular basis of male fertility remains unclear, especially in chickens, where decades of genetic selection induced an important increase in male fertility variability as a side effect (Soler et al., 2016a). To determine the crucial proteins involved in sperm-fertilizing abilities in order to decipher the impaired molecular pathways in subfertile animals and potentially link them to semen defects as causes of subfertility, *in vivo* rooster fertility was determined by AI in order to evaluate sperm-fertilizing ability and define animals according to their fertility status, i.e., fertile (FR > 70%) or subfertile (FR < 40%). Semen from F and SF roosters were then collected and *in vitro* characterized, and sperm protein content was analyzed by two complementary proteomic approaches, showing important candidates involved in the variations in sperm fertilizing ability.

In various species, sperm characteristic is evaluated by classical *in vitro* tests (WHO, 2010), evaluating semen volume, concentration, mass motility, and individual cell motility. Here, these tests revealed that the observed fertility reduction in some individuals of meat-type roosters was mainly associated with a decrease of mass motility, whereas the individual sperm motility analysis by CASA was not impacted (Figure 1). However, the small non-significant reduction of percentages of motile, rapid, and progressive sperm obtained by individual investigation may, at the scale of the whole ejaculate, result in the observed reduction of mass motility. Thus, small differences of sperm cell motility at the individual scale may result to a significant reduction of the whole sperm cell movement of the ejaculate, leading to a reduction of male fertility. In sheep, the association of mass motility reduction and impaired fertility has been reported (David et al., 2015). In addition to the individual movement of each sperm cell, the collective progress of sperm cells also appeared necessary to successfully reach the oocyte. So, our data revealed that, at least, one of the causes of subfertility in meat-type roosters was relative to sperm motility defects. However, we showed also that other sperm functions are affected.

To explore differences in sperm protein content between F and SF roosters, we used two complementary proteomic approaches: the ICM-MS and the GeLC-MS/MS. Indeed, whereas ICM-MS focused on the identification of small proteins and peptidoforms, as a result of protein degradation, without protein isolation, GeLC-MS/MS determines larger proteins previously extracted and digested. In contrast to GeLC-MS/MS (Supplementary Data 3), the ICM-MS approach succeeded in segregating F and SF animals on the basis of 211 detected peaks (Figure 2). Interestingly, PCA analysis revealed a more important variability within SF animals than within F animals, suggesting that causes of fertility reduction in SF roosters were multiple, corresponding to various proteomic profiles, as previously described (Labas et al., 2015). Furthermore, our data revealed that three of the differentially abundant peaks revealed by ICM-MS correspond to peptidoforms of CFAP100, TUBB7, and ENO1 (whose functions are described later), all more abundant in SF animals than in F roosters (Figure 2). These products certainly result from proteolytic events which could only be identified by ICM-MS methodology, known for highlighting specific or unspecific protease activities generally unrevealed by classical proteomics (Labas et al., 2018). Consequently, an increase of proteolytic events could be assumed in SF sperm, leading to defects on various molecular pathways involved in sperm-fertilizing ability. Moreover, the origin of the peptidoforms identified by ICM-MS remains a challenge, because of the limitation of exhaustive top-down MS databases for exhaustive proteome, as shown in our study by the absence of annotation of other differentially abundant peaks (Figure 2). Thus, whereas ICM-MS appeared to be limited to develop an exhaustive list of proteins involved in sperm-fertilizing abilities, it remains an efficient and sensitive methodology for the identification of small proteins and larger protein degradation products, using small amounts of sperm cells (∼10^5^–10^6^ cells/spot) without prior sample preparation, making the ICM-MS approach a promising test to evaluate quality semen and diagnose male fertility, as previously described (Soler et al., 2016a, 2020).

In order to increase the list of DAP involved in chicken sperm-fertilizing abilities, we therefore used the GeLC-MS/MS method and identified 54 DAP between roosters of the two fertility status (Figure 3 and Table 1), increasing the list of DAP involved in meat-line rooster sperm-fertilizing ability to 57 proteins. For most of them (at the exception of tubulins, tektins, and ovotransferrin), this data is the first report of their involvement in rooster fertility (Labas et al., 2015; Soler et al., 2016a). The difference of abundance of 2 DAP, one more abundant in F animals—ovotransferrin (TF)—and another more abundant in SF roosters—lysozyme (LYZ), was confirmed by immunoblot (Figure 4 and Table 2). This finding underlines the complementariness of our combined approach associating ICM-MS and classical bottom-up proteomics (Labas et al., 2015) for characterization of markers. Of note, both label-free quantitative proteomic approaches revealed LYZ as significantly more abundant in SF animals when compared to F roosters (Figures 2–4 and Table 2), and immunoblot confirmed the robustness of these quantifications. Altogether, these DAP reflect major dysregulations of flagellum integrity and functions, mitochondria activity, sperm maturation, and cellular interaction.

As a direct reflect of the observed reduction of mass motility, the axoneme integrity appeared impacted by the deregulation of TUBB (beta tubulins) and TEKT (tektins) abundances, crucial components of this cytoskeletal structure forming the flagellum core (Popodi et al., 2005; Raff et al., 2008). Whereas TEKT absence leads to a reduction of sperm motility in mice (Roy et al., 2007, 2009), the increase of TUBB and TEKT family members in SF sperm may increase the flagellum rigidity, also leading to a reduction of sperm motility. Interestingly, a perturbation of TEKT in SF animals was already reported in previous studies (Labas et al., 2015; Soler et al., 2016a), confirming the importance of these proteins in chicken sperm-fertilizing ability. However, total variation TUBB abundance may not be the only cause of TUBB influence on axoneme formation, since an increase of proteolytic regulation of TUBB7 has been revealed by ICM-MS experiments (Figure 2). Furthermore, multiple members of the TCP/CCT family were less abundant in SF sperm and these proteins are known to be involved in the cytoskeleton integrity by modeling of newly synthetized actin and tubulin monomers (Brackley and Grantham, 2011). Consistently with our data, reduction of their expression is associated with sperm motility decrease (Castaneda et al., 2020). Whereas not revealed in sperm cells, the presence of CFAP100 (cilia- and flagella-associated protein 100) is crucial for the ciliary movement of *Chlamydomonas reinhardtii*, as involved in the regulation of axoneme assembly (Yamamoto et al., 2013). A similar function could be suggested in chicken sperm, and the degradation of CFAP100 by proteolysis may impair the flagellum movement. In addition to the axoneme constitution, the fibrous sheath composition and activity also appeared impaired, especially with the dysregulation of proteins relative to local energy production like ALDOA (aldolase A), HK3 (hexokinase 3), and DYL2 (dynein light chain 2). ALDOA and HK3 would be involved in the flagellum glycolysis process needed for local ATP production (Krisfalusi et al., 2006; Nakamura et al., 2008), and DYL2 in the response of the CA^2+^ wave responsible for flagellum beating (Mizuno et al., 2012). Similarly, ENO1, present in the tail of sperm cells, is a glycolytic enzyme regulating enzyme activity to produce the energy in microtubules and to protect male gametes against oxidative stress (Park et al., 2013, 2019). ALDOA, HK3, and DYL2 were less abundant in SF animals, and ENO1 was more degraded in SF roosters, suggesting a reduction in local energy for flagellum movement and beating, and is consistent with the observed reduction of mass motility in SF individuals. PCA analysis suggests a positive correlation between mass motility and ALDOA, TCP1, DYL2, and HK3 (Figure 3). However, correlation analysis implies large sample size (Bujang and Baharum, 2016) and, thus, these putative correlations should be validated on a larger sample size, by immunoblot for instance.

**FIGURE 5:**
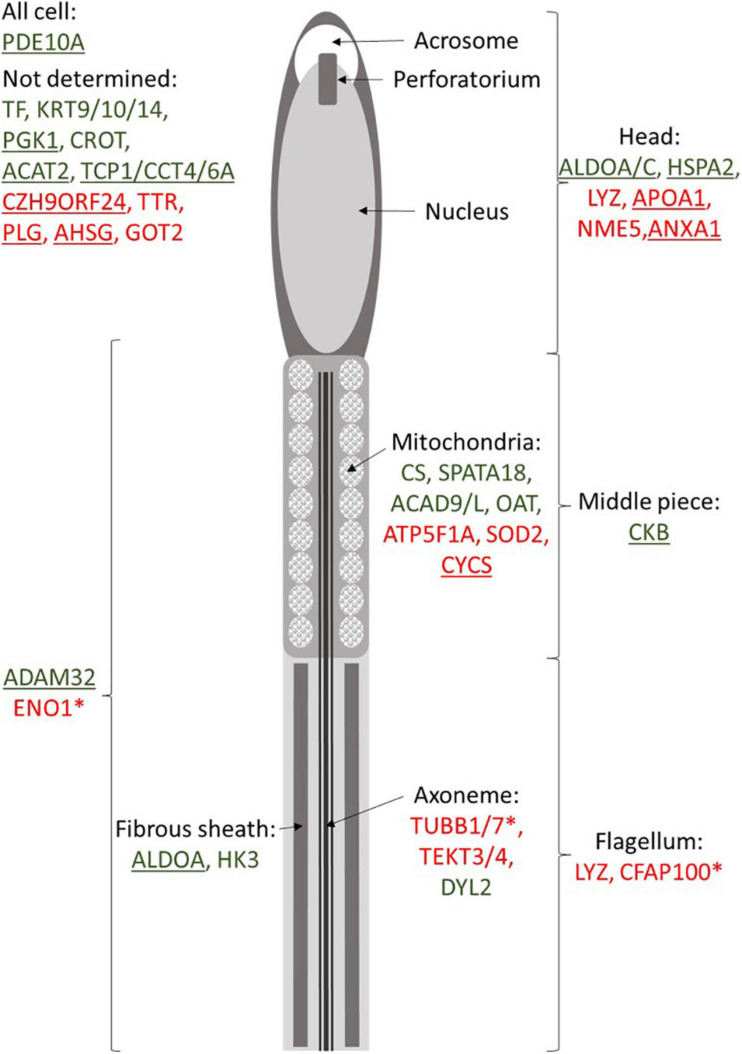
Putative localization of proteins identified as relative to meat-line rooster fertility. Peptidoforms resulting from protein degradation revealed by ICM-MS are noted with asterisks. Green indicates proteins more abundant in fertile animals, and red represents proteins and peptidoforms more abundant in subfertile roosters. Underlined proteins are the one present in chicken seminal plasma (Labas et al., 2015).

Our data also revealed numerous DAP involved in mitochondria integrity and activity such as CS, SPATA18, ACAD9/L, OAT, ATP5A1, SOD2, and CYCS (Figure 5). Some of these actors, such as CYCS (cytochrome C) or SOD2 (superoxide dismutase 2), are known to be involved in the oxygen stress response of sperm cells (Wang et al., 2003; Yan et al., 2014). High abundance of OAT (ornithine aminotransferase) and ATP5F1A (ATP synthase F1 subunit alpha) has been reported in high-quality sperm in bovine and human (D’Amours et al., 2019; Panner Selvam et al., 2019). Obviously, impairment in mitochondria integrity and activity may impact the whole-cell physiology, including its motility. Several proteins involved in the energy transduction, such as CKB (creatine kinase B-type), was less abundant in SF roosters. The abundance of CKB is linked with sperm cell maturity in mammals (WHO, 2010), and its abundance is linked to sperm motility in mammals (Parte et al., 2012) and fish (Dietrich et al., 2016). This finding underlines the importance of energy transduction for chicken sperm functions, and not only the energy production.

Interestingly, proteomic approaches developed here also revealed dysregulations in molecular pathways linked to events occurring in the female tracts, and not accessed by classical *in vitro* semen quality evaluation, as performed here. For instance, our data revealed a higher level of ANXA1 (annexin A1) in SF animals. The presence of ANXA1 has been reported in seminal plasma in mammals (Candenas and Chianese, 2020), and in chickens (Labas et al., 2015; Li et al., 2020). Sperm surface annexins have been associated with the adhesion of sperm to oviductal cells in mammals in order to hold sperm in oviductal reservoir (Ignotz et al., 2007; Teijeiro et al., 2009). In chickens, sperm storage into the female tract occurs during weeks within sperm storage tubules (King et al., 2002). Thus, the reduction of ANXA1 in SF sperm may sign the reduction of sperm capacities to be stored into the female tract. Furthermore, our data reported a high amount of APOA1 in SF animals. APOA1 has been reported in chicken seminal plasma in higher quantity in SF animals (Labas et al., 2015), suggesting a differential impact of seminal plasma in sperm maturation. In mammals, APOA1 is present in the sperm head and known to induce cholesterol efflux in spermatozoa necessary for sperm capacitation (Kwon et al., 2014). The modification of the APOA1 amount in chicken sperm in relation with the fertility suggests a sperm maturation deregulation. Moreover, in humans, a decline of sperm motility was observed when sperm APOA1 was inhibited by the presence of antibodies (Chi et al., 2018), suggesting a decrease of sperm motility subsequently to the reduction of sperm maturation. Finally, multiple proteins are known to be involved in the oocyte–sperm interaction in mammals such as the chaperone HSPA2 (Redgrove et al., 2012), LYZ (Herrero et al., 2005; Sun et al., 2011), and ADAM32 (Vicens et al., 2014). Thus, recent studies described that LYZ is present in the sperm cell surface and that its neutralization in male rats induces male infertility, by impairing acrosome reaction (Sun et al., 2011; Narmadha and Yenugu, 2016). Actually, LYZ abundance observed by immunoblot was not statistically correlated with any of the sperm quality parameters tested *in vitro* (Supplementary Data 4), which were all indicators of motility abilities and not of the acrosome integrity. Testing the acrosome functionality *in vitro* is challenging in chicken sperm (Lemoine et al., 2008) and not classically performed to evaluate semen quality. In this context, it would be interesting to investigate if LYZ abundance could represent an indirect measure of acrosome performance. Additionally, whereas not directly involved in oocyte–sperm interaction, PDE10 is essential to acrosome reaction and thus to oocyte–sperm fusion in mammals (Maréchal et al., 2017). Similar functions could be expected in chicken sperm. Another example with the mammalian equivalent of TF, the lactoferrin (LT), has been reported to be expressed by the epididymis, capable of sperm binding (Jin et al., 1997), increasing the membrane functionality of cryopreserved sperm (Martins et al., 2018), and modulating the oocyte–sperm interaction (Zumoffen et al., 2015). Thus, similar functions could be assumed for avian equivalent, the TF, which may also be involved in the oocyte–sperm interaction, and its reduction in sperm from SF roosters may sign a putative dysregulation of oocyte recognition by sperm cells in SF animals. Further investigations will be necessary to explore the role of these proteins in oocyte–sperm interaction in chicken.

Collectively, our data revealed that the fertility reduction observed in some meat-type roosters may be partly related to the decrease of sperm mass motility. However, among the 50 proteins newly revealed by the developed proteomic strategies and linked for the first time to the chicken fertility status, not all appeared directly relative to motility defects. In fact, other molecular pathways seemed to be impaired, including mitochondria functions, sperm maturation, and events occurring into the female tract, including storage as well as oocyte–sperm interaction. Therefore, our data confirmed the complexity of sperm-fertilizing capacity, a phenomenon not classically revealed by *in vitro* semen quality evaluation. Thus, it is crucial to obtain a large overview of the sperm proteome at the individual level to decipher the maximum of the mechanisms involved in sperm-fertilizing capacity. Here, we showed that ICM-MS and GeLC-MS/MS approaches were complementary to discriminate animals according to their fertility status based on multiple markers (peptidoforms and proteoforms) and to identify impairments into sperm biological pathways, respectively. Among the proteins identified here, some, such as ENO1, are also known as fertility markers in bull sperm cells (Park et al., 2013, 2019). Thus, these data are thus highly valuable to better understand the molecular processes involved in chicken sperm-fertilizing abilities, but also in the identification of putative robust male fertility biomarkers shared by different animal species.

## Data Availability Statement

The datasets presented in this study can be found in online repositories. The names of the repository/repositories and accession number(s) can be found in the article/Supplementary Material.

## Ethics Statement

All experiments were carried out in accordance with the European welfare and the French Direction of Veterinary Services regulations (agreement number C37–175-1) as previously described.

## Author Contributions

EB designed the research. LS, AT, IG, LC, A-PT-G, and VL performed the research. AVC, DT, EB, and VL analyzed the data. AVC, LS, VL, and EB wrote the manuscript. All authors contributed to the article and approved the submitted version.

## Conflict of Interest

The authors declare that the research was conducted in the absence of any commercial or financial relationships that could be construed as a potential conflict of interest.

## Footnotes

1 https://doi.org/10.15454/1.5572326250887292E12

## Supplementary Material

Supplementary Data 1 ICM-MS differential analysis. Each mass represents one peak and the difference between fertile (F) and subfertile (SF) animals (*n* = 9) was by non-parametric Mann-Whitney or Wilcoxon test to determine the *p*-value.

Supplementary Data 2 DAP revealed by GeLC-MS/MS included in PCA analysis. Only DAP with a gene description and a gene symbol not corresponding to LOC were included.

Supplementary Data 3 PCA analysis of GeLC-MS/MS. PCA analysis was conducted based on all the list of 84 DAP revealed by GeLC-MS/MS, with a preferential selection of SC differential.

Supplementary Data 4 Correlation analysis of western-blot abundance quantifications of ovotransferrin (WB_TF) and lyzosyme (WB_LYZ), fertility rate and *in vitro* semen quality evaluation parameters. Square including dot presents significant correlation (*p* < 0.05). Dot color and intensity corresponds to correlation direction and strength, respectively.
