# Supplementary figures and images for: Proteomic Changes Associated With Sperm Fertilizing Ability in Meat-Type Roosters
Source: Front Cell Dev Biol. 2021 Apr 9;9:655866. doi: 10.3389/fcell.2021.655866 (PMC8063615; doi:10.3389/fcell.2021.655866)

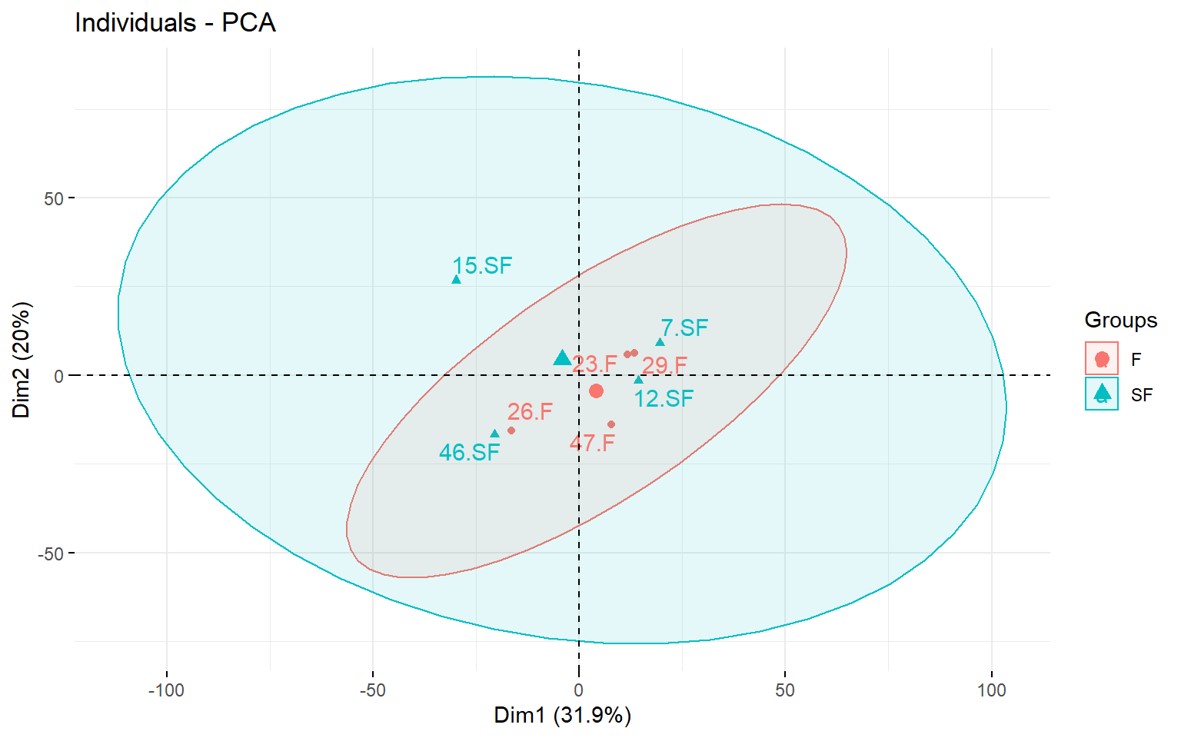

Supplement: Supplementary file 5 [file Image_1.JPEG]

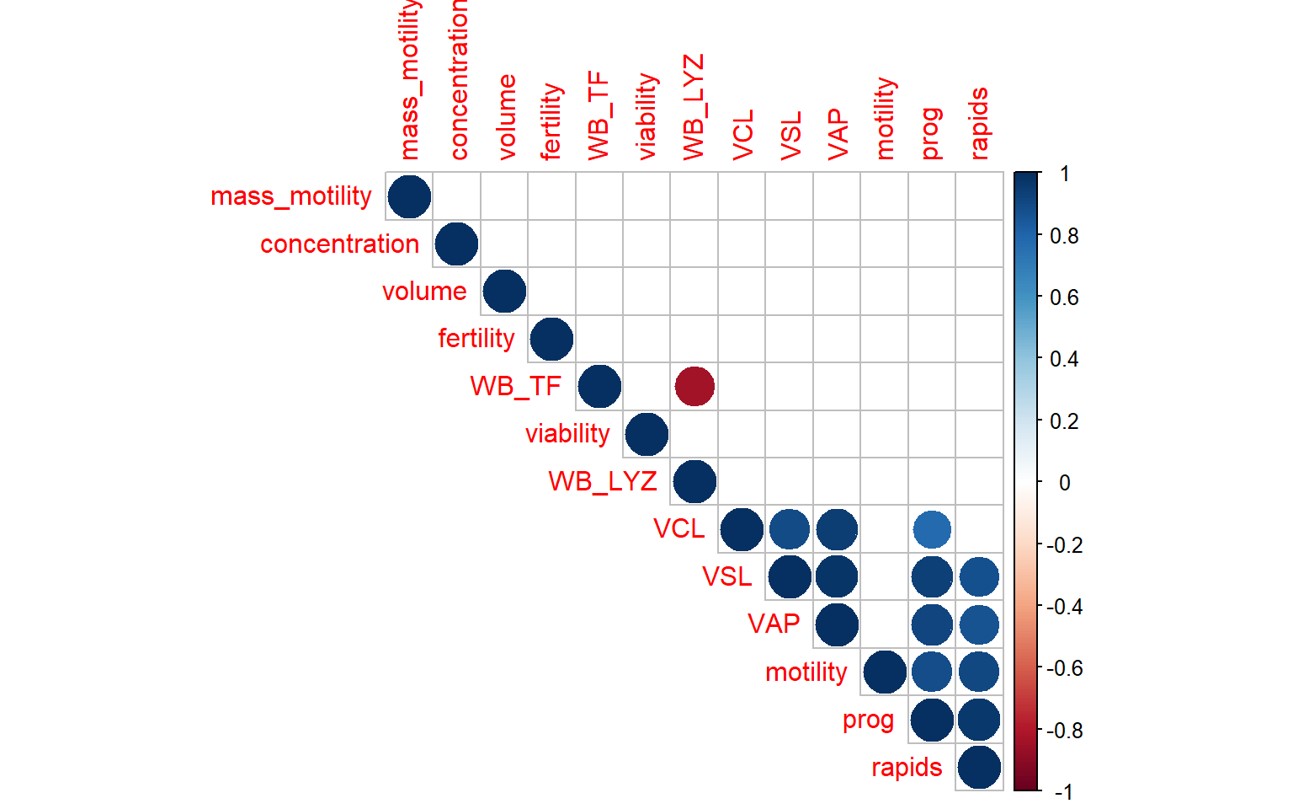

Supplement: Supplementary file 6 [file Image_2.JPEG]
